# Endotherapy for small-bowel recurrent bleeding from a jejunal cavernous hemangioma in an elderly patient

**DOI:** 10.1055/a-2346-4938

**Published:** 2024-07-08

**Authors:** Noemi Gualandi, Pablo Cortegoso Valdivia, Giuliano Francesco Bonura, Paola Soriani, Mauro Manno

**Affiliations:** 1 Gastroenterology and Digestive Endoscopy Unit, Azienda USL Modena, Carpi, Italy; 2 Gastroenterology and Endoscopy Unit, University Hospital of Parma, University of Parma, Parma, Italy

An 83-year-old man, with a history of hypertension, diabetes, and chronic liver disease, presented to the emergency room with fatigue and melena. Blood tests revealed iron-deficiency anemia (Hb 6.5 g/dL). Esophagogastroduodenoscopy (EGD) showed a Forrest III ulcer in the gastric antrum and a small, nonbleeding gastric angiodysplasia, which was treated with argon plasma coagulation. As colonoscopy was unremarkable, the patient was discharged after a few days.

The patient was readmitted 3 months later for recurrence of bleeding. Repeat EGD was negative for bleeding lesions; therefore, capsule endoscopy was performed in <48 hours. Capsule endoscopy showed a 5-mm ulcerated polyp with an adherent clot in the jejunum (Saurin P2 lesion) ( Fig. 1 ). A push enteroscopy (SIF-H190; Olympus, Tokyo, Japan) was then performed, confirming the finding of a sessile polyp in the mid jejunum ( Fig. 2 ), which was removed en bloc with a braided snare after submucosal injection. Finally, the base was prophylactically closed with hemoclips ( Fig. 3 , Video 1 ).

**Fig. 1:**
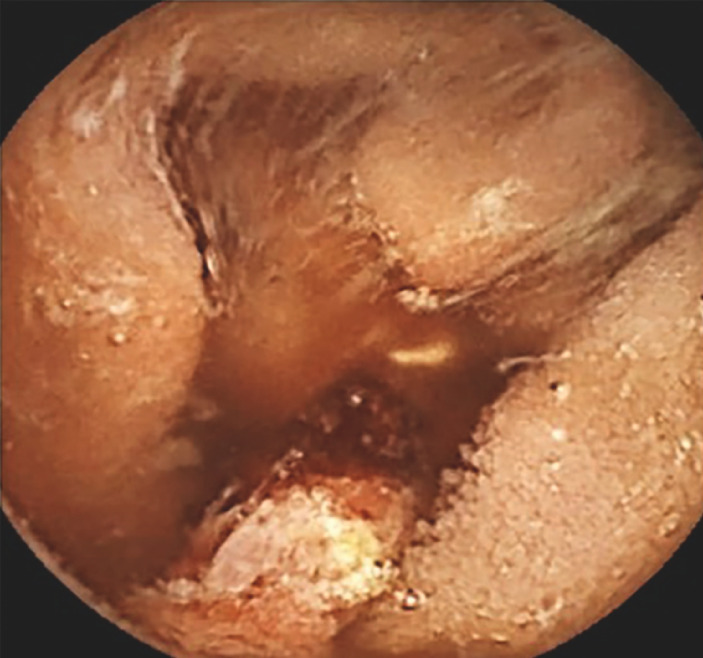
Capsule endoscopy detected a jejunal P2 lesion (Saurin classification).

**Fig. 2:**
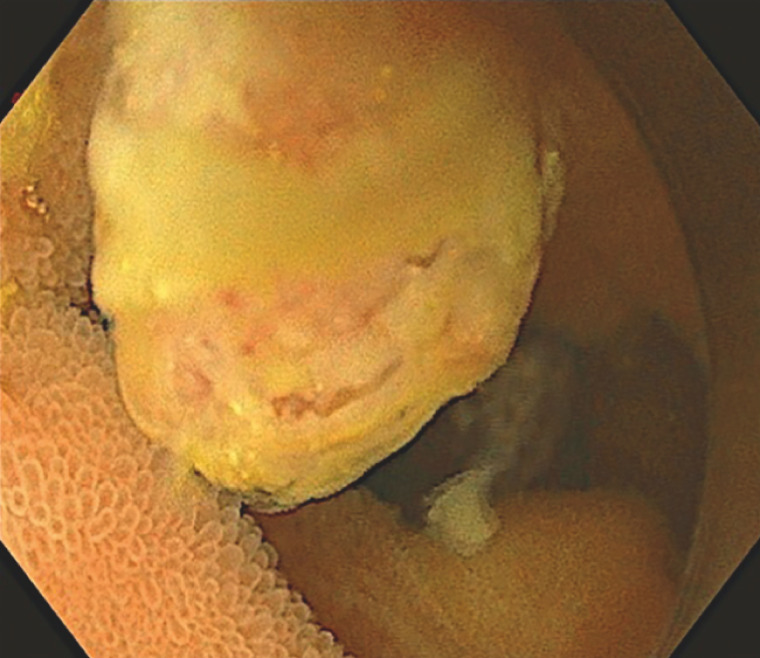
Enteroscopic image of the ulcerated 5-mm polyp in the mid jejunum.

**Fig. 3:**
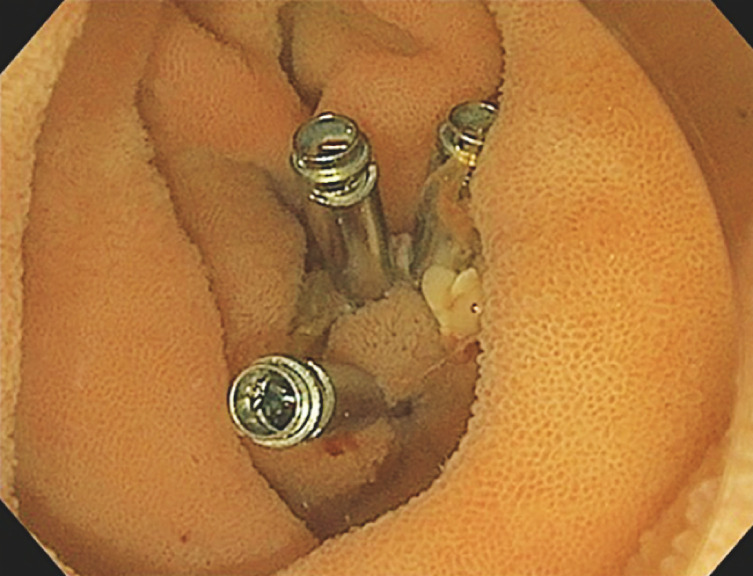
The cutting base was closed with hemoclips after mucosal resection.

Capsule endoscopy diagnosis and enteroscopic resection of a jejunal cavernous hemangioma in a patient with recurrent bleeding episodes.Video 1

Histology of the specimen showed jejunal nondysplastic ulcerated mucosa, with vascular proliferation and dilation of the capillaries ( Fig. 4 ). The results were consistent with the diagnosis of cavernous hemangioma.

**Fig. 4:**
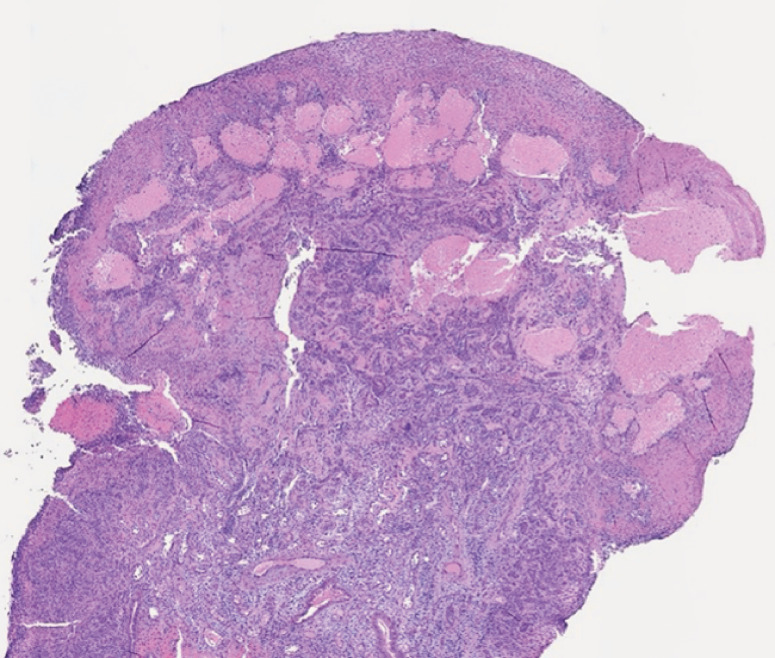
Histological diagnosis of cavernous hemangioma.

Cavernous hemangiomas are rare vascular malformations of mesenchymal origin, potentially involving the small bowel and accounting for 7%–10% of all benign tumors in this gastrointestinal segment 1 2 3 . Similarly to other benign small-bowel tumors, cavernous hemangioma may remain asymptomatic for many years before becoming clinically manifest, usually with iron-deficiency anemia or with gastrointestinal bleeding (either overt or occult, often intermittent) 4 5 . Although small-bowel cavernous hemangioma is mostly common in the young, elderly patients may also be affected 1 3 .

At a 3-month follow-up, the patient remained asymptomatic with no bleeding recurrence, showing that enteroscopic resection is a safe therapeutic option for cavernous hemangioma.

Endoscopy_UCTN_Code_TTT_1AP_2AD

## Notes

Endoscopy E-Videos https://eref.thieme.de/e-videos
*E-Videos* is an open access online section of the journal *Endoscopy* , reporting on interesting cases and new techniques in gastroenterological endoscopy. All papers include a high-quality video and are published with a Creative Commons CC-BY license. Endoscopy E-Videos qualify for HINARI discounts and waivers and eligibility is automatically checked during the submission process. We grant 100% waivers to articles whose corresponding authors are based in Group A countries and 50% waivers to those who are based in Group B countries as classified by Research4Life (see: https://www.research4life.org/access/eligibility/ ). This section has its own submission website at https://mc.manuscriptcentral.com/e-videos .
